# Measurement of health-related quality by multimorbidity groups in primary health care

**DOI:** 10.1186/s12955-018-1063-z

**Published:** 2019-01-11

**Authors:** Magdalena Millá-Perseguer, Natividad Guadalajara-Olmeda, David Vivas-Consuelo, Ruth Usó-Talamantes

**Affiliations:** 1 Health district Valencia-Hospital General, Conselleria de Sanitat Universal i Salut Pública. Generalitat Valenciana, Valencia, Spain; 2 Centre of Economic Engineering, Universitat Politècnica de València, Valencia, Spain; 3 Health district Valencia Clínico-Malvarrosa, Conselleria de Sanitat Universal i Salut Pública. Generalitat Valenciana, Valencia, Spain

## Abstract

**Background:**

Increased life expectancy in Western societies does not necessarily mean better quality of life. To improve resources management, management systems have been set up in health systems to stratify patients according to morbidity, such as Clinical Risk Groups (CRG). The main objective of this study was to evaluate the effect of multimorbidity on health-related quality of life (HRQL) in primary care.

**Methods:**

An observational cross-sectional study, based on a representative random sample (*n* = 306) of adults from a health district (*N* = 32,667) in east Spain (Valencian Community), was conducted in 2013. Multimorbidity was measured by stratifying the population with the CRG system into nine mean health statuses (MHS). HRQL was assessed by EQ-5D dimensions and the EQ Visual Analogue Scale (EQ VAS). The effect of the CRG system, age and gender on the utility value and VAS was analysed by multiple linear regression. A predictive analysis was run by binary logistic regression with all the sample groups classified according to the CRG system into the five HRQL dimensions by taking the “healthy” group as a reference. Multivariate logistic regression studied the joint influence of the nine CRG system MHS, age and gender on the five EQ-5D dimensions.

**Results:**

Of the 306 subjects, 165 were female (mean age of 53). The most affected dimension was pain/discomfort (53%), followed by anxiety/depression (42%). The EQ-5D utility value and EQ VAS progressively lowered for the MHS with higher morbidity, except for MHS 6, more affected in the five dimensions, save self-care, which exceeded MHS 7 patients who were older, and MHS 8 and 9 patients, whose condition was more serious. The CRG system alone was the variable that best explained health problems in HRQL with 17%, which rose to 21% when associated with female gender. Age explained only 4%.

**Conclusions:**

This work demonstrates that the multimorbidity groups obtained by the CRG classification system can be used as an overall indicator of HRQL. These utility values can be employed for health policy decisions based on cost-effectiveness to estimate incremental quality-adjusted life years (QALY) with routinely e-health data.

Patients under 65 years with multimorbidity perceived worse HRQL than older patients or disease severity. Knowledge of multimorbidity with a stronger impact can help primary healthcare doctors to pay attention to these population groups.

## Background

The population in Spain has significantly aged in recent years due to lower fertility rates and higher survival rates thanks to better treatments for potentially lethal diseases [1]. Life expectancy today in Spain is 83.3 years, which is the highest of all EU countries [2]. However after reaching the age of 65, some form of disability is suffered for half the years left to live. Here disability is understood as a generic term that covers impairments, activity limitations and participation restrictions (according to the Classification of Functioning, Disability and Health) [3]. This spells a bigger demand for health services and long-term care [2].

Most health problems that affect the elderly are associated with either chronic diseases (health problems that need to be followed up for several years or decades) or multimorbidity (having two chronic health situations or more) [4, 5].

Chronic conditions are also a main cause of death (60%) in almost all developed countries [6]. Their prevalence increases and their relation with age is reflected in several studies [7–9]. Nowadays, however, almost half of deaths from chronic diseases occur in subjects who are not yet 70 years of age, and one fourth occur in people under the age of 60. Some studies even indicate that multimorbidity prevalence increases in young adults. Thus multimorbidity is not limited to old age [5, 10–15].

Given the need to quantify multimorbidity, risk-adjusted systems in health have been developed worldwide. One of the most widely used systems is the Clinical Risk Groups (CRG) systems [16], which have been implemented in the Valencian Community (Spain). The CRG system includes clinical morbidity data from electronic health records, primary-care and hospital procedures, and demographic characteristics, and others can be added, such as pharmaceutical utilisation. This system uses all the diagnosis codes in the International Classification of Diseases (ICD.9.MC and/or ICD.10) assigned to each person for about 1 year. It also includes records from any health care service level. Thus each individual is assigned a mutually excluding CRG. At a first level, the population is classified into 1075 groups (version 1.6), but each CRG category is grouped into three aggregated classifications level. The most aggregated level comprises nine Main Health Statuses (MHS). These nine MHS are: 1 Healthy; 2 History of significant acute disease; 3 Single minor chronic disease; 4 Minor chronic diseases in multiple organ systems; 5 Significant chronic disease; 6 Significant chronic diseases in multiple organ systems; 7 Dominant chronic disease in three organ systems or more; 8 Dominant/Metastatic malignancy and 9 Catastrophic. In practical terms, CRG can forecast and explain health expenditure [14–18], which allows resources to be better assigned according to morbidity and population requirements.

The WHO defines quality of life as individuals’ perception of their position in life in the context of the culture and value systems in which they live, and in relation to their goals, expectations, standards and concern [19]. Increased life expectancy in Western societies where medicine, apart from the role it plays, must be that which confers people many years of life by providing improved quality of life in the years they have left to live, and this leads us to the health-related quality of life (HRQL) concept [20]. Previously, Revicki [21] defined HRQL as a multidimensional concept that encompasses the physical and socio-emotional components associated with a disease or treatment. The HRQL measure has been used as a health indicator when studying and assessing health procedures [22, 23].

It is difficult to measure HRQL given primary-care practitioners’ working conditions, lack of time and extremely wide-ranging patients with vastly differing socio-demographic characteristics and very many distinct health problems. One of the most widely used HRQL measuring instruments is EQ-5D [24]. It use in primary-care offers the advantages of conducting a very short, low cognitively-demanding questionnaire that is simple to apply and takes 2 or 3 min to administer. Its validity has been verified in several diseases and it has proven sensitive to changes in health statuses in several patient groups [25]. It is a simple generic standard measure of health statuses developed by the EuroQol group for clinical and economic evaluations [26]. The EQ-5D comes in two versions, 3-level (EQ-5D-3 L) and 5-level (EQ-5D-5 L), and the properties in relation to multimorbidity of both are similar [27].

In Spain, one of the National Health System Quality Plan objectives is to implement strategies that ensure all citizens maximum health care quality. This health care is carried out in a health system with a public financing service and comprehensive coverage. Primary-care is the first level where health is promoted, disease is prevented and rehabilitation measures are offered [28]. In this context, the HRQL measure is an indicator by which patients express their views and perceptions about their health status, which measures the effect that health care or its disease evolution process has on them. This means that the impact that a disease has on a patient’s day-to-day life is obtained [29]. The Spanish National Health Survey 2011/12 [30], conducted by the Spanish Ministry of Health, Social Services and Equality since 2003 in collaboration with the Spanish National Statistics Institute (INE), includes a report with the HRQL results of an adult population obtained with the EQ-5D questionnaire. The EQ-5D results provide a populational control for Spain, which is representative for Spanish Autonomous Communities. It acts as a reference to compare patient groups and the general population, and to follow-up patients’ health evolution and, thus, contributes to obtain effectiveness measures to assess health technologies. The report describes the HRQL of the Spanish population aged over 18 years [30].

Very few studies have included HRQL and multimorbidity [11, 22, 31, 32], and those that have do not map utility values with multimorbidity groups. However, other studies map [33–35] routine clinical outcome measures of health to a health state utility value generic instrument, such as EQ-5D. This means that these utility values can be used to make economic evaluations to estimate cost per quality-adjusted life year (QALY).

The main objective of this study was to evaluate the effect of multimorbidity on HRQL in the primary-care context. We used the main CRG aggregated groups as a measure of multimorbidity.

## Materials and methods

### Design

This is a cross-sectional study conducted in a health district in the Valencian Community. The Valencian Community is located in east Spain. It has a population of 5,155,000 and includes 24 health areas that offer integrated health care and form part of the Spanish National Health System. This health district encompasses four primary health centres with 17 GPs and seven paediatricians. This study included all the citizens registered in this health district with a doctor assigned since December 2013 (*N* = 32,667).

### Sources of information

Data were obtained from the electronic health records and administrative health data included in the following information systems: the population information system, the primary-care information system, the minimum database series, the electronic pharmaceutical prescription system and the Valencian Community patients classification system. All these systems are centralised in a single e-database that belongs to the Regional Valencian Ministry of Universal Health and Public Health of the Valencian Community. The population was stratified using version 1.6 of the 3M™ Clinical Risk Grouping Software, which employs diagnosis coding by ICD9-MC.

The population was stratified into nine groups, MHS. The first three groups are considered acute processes, while MHS 4 onwards form the populations with chronic conditions or multimorbidity. The HRQL data were obtained with a survey conducted with a random population sample, which we go on to explain.

### Study sample

The sample was recruited, based on the random selection of patients stratified according to their MHS group. As we had no prior references, sample size was calculated with the most unfavourable *p*-value: 0.5 (50%), with a confidence level of 95% and a 4% sampling error [36]. The whole sample included 882 individuals. We performed stratified random sampling with equal sized strata [31] so that all the MHS would be equally represented in the models, and not biased in favour of the more numerous ones. This gave 98 individuals per MHS.

The exclusion criteria were: aged under 18 years, no contact by telephone, cases who left the research during the study period, change of residence, patients with cognitive alterations and patients hospitalised in social institutions. Nonetheless, given the small population size classified in group MHS 8 (99 patients) and MHS 9 (103 patients) (see Table 1), it was possible to only obtain a response in 33 patients and 32 patients, respectively. This conditioned the size of the other MHS, and led to a final sample with 306 patients and to a 5.7% sampling error.

**Table 1:** Distribution of the health district population and age. Distribution of the sample, age, gender, the TTO score and EQ VAS for the nine MHS

	Population		Sample				
Clinical risk groups main health statuses	N 32,667 N%		n 306	Males 46% mean age (years)	Females 54% mean age (years)	EQ 5D 3 L TTO score mean 95%CI	EQ VAS mean 95%CI
1. Healthy	17,601	53.9	50	41 (30)^a^	36 (29)^a^	0.93 [0.90, 0.96]	80.32 [75.94, 84.70]
2. History of significant acute disease	1696	5.2	40	32 (32)^a^	36 (33)^a^	0.87 [0.79, 0.94]	69.75 [63.73, 75.77]
3. Single minor chronic disease	3487	10.7	30	46 (41)^a^	44 (41)^a^	0.81 [0.70, 0.92]	65.83 [57.69, 73.98]
4. Minor chronic diseases in multiple organ systems	1333	4.1	31	56 (55)^a^	55 (54)^a^	0.77 [0.69, 0.85]	66.45 [61.73, 71.17]
5. Significant chronic disease	4803	14.7	30	47 (47)^a^	53 (51)^a^	0.75 [0.63, 0.86]	62 [54,27, 69,73]
6. Significant chronic diseases in multiple organ systems	3201	9.8	30	56 (64)^a^	65 (70)^a^	0.58 [0.46, 0.70]	55.83 [48.73, 62.94]
7. Dominant chronic disease in 3 or more organ systems	344	1.1	30	74 (75)^a^	77 (79)^a^	0.60 [0.48, 0.72]	57 [46.67, 67.33]
8. Dominant/Metastatic malignancy	99	0.3	33	67 (64)^a^	64 (64)^a^	0.59 [0.46, 0.73]	51.67 [44.01, 59.33]
9. Catastrophic	103	0.3	32	49 (48)^a^	52 (51)^a^	0.49 [0.33, 0.64]	53.13 [43.02, 63.23]

*TTO score* Time Trade-Off score

*EQ VAS* EQ Visual Analogue Scale

*CI* confidence interval

^a^mean population age in brackets

The HRQL questionnaire was conducted by telephone as it can be easily applied. The evaluation of their HRQL was obtained with their responses, which complies with personal data protection regulations.

### Measuring instruments and interventions

EQ-5D-3 L was applied to the sample, which essentially consists of two pages: the EQ-5D descriptive system and the EQ Visual Analogue Scale (VAS). The EQ-5D-3 L descriptive system comprises the following five dimensions: mobility, self-care, usual activities, pain/discomfort and anxiety/depression. Each dimension has three levels of severity: no problems, some problems, and extreme problems. The patient is asked to indicate his/her health state by ticking the box next to the most appropriate statement in all five dimensions. This decision results in a 1-digit number that expresses the level selected for that dimension. The digits for the five dimensions can be combined into a 5-digit number that describes the patient’s health status.

By combining the five dimensions and the three severity levels, 243 possible health state patients’ QoL were obtained. These health states of patients were converted into a single value by applying the Time Trade-Off (TTO) method, in which each dimension has set weights or utility values required for economic evaluations [25]. This index was calculated by deducting the value obtained with the previous formula from 1, which ranges between 1 (a better HRQL, 11111) and 0 (a worse HRQL or death). It provides a value of an individual’s preference to live the rest of his/her life with the current HRQL, as opposed to living less time, but with an excellent HRQL [37, 38]. We used the Spanish TTO value set to obtain these utility values or the TTO score [39].

The EQ VAS records the patient’s self-rated health on a vertical visual analogue scale where endpoints are labelled ‘Best imaginable health state’ (value 100) and ‘Worst imaginable health state’ (value 0). The EQ VAS can be used as a quantitative measure of health outcome that reflects the patient’s own judgement.

### Statistical analysis

A descriptive analysis was done of both the study population and sample according to age, gender and the nine MHS. The following were also analysed: the TTO score, EQ VAS and the five dimensions of the sample by the nine MHS.

Different analyses were carried out to know the influence of the CRG system, age and gender on the perception of HRQL. Firstly, multiple linear regression adjusted by ordinary least squares was done [40] and several models were obtained, where the dependent variables were: the TTO score and EQ VAS. The independent or explanatory variables were: the nine MHS (dummy variables, where MHS 1, “healthy,” was the reference group), age (continuous variable) and gender (variable dummy, 0 = male and 1 = female). We obtained the beta coefficients of each variable as a measurement of the mean effect size and the adjusted R^2^ value, which provided us with the percentage of variance of the dependent variable explained by the set of independent variables.

Secondly, a predictive analysis was run with all nine MHS on the five EQ-5D-3 L dimensions by binary logistic regression. To this end, all five health dimensions were considered a dependent variable by grouping severity levels 2 (some problems) and 3 (extreme problems) with a value of “1”, and by assigning severity level 1 (“no problems”) a value of “0”. In this way, each dimension was quantified in a binary form [22]. We obtained the exponential of the beta coefficient, the equivalent to the Odds Ratio. We had to transform the values below 1 into their inverse to be able to compare them.

Thirdly, multivariate logistic regression was done to explain all five EQ-5D-3 L dimensions in a binary manner according to the nine MHS, age and gender [41]. MHS 1 was taken as the reference group.

Version 16.0 of the SPSS Statistical Package and Microsoft Excel 2012 were employed for the data analysis.

## Results

The health district population’s mean age was 39.2 years. Table 1 provides its distribution into multimorbidity groups.

Of the 306 subjects who formed the study sample, 46% (*n* = 141) were male and 54% (*n* = 165) were female, and their mean age was 52 and 54 years, respectively. The MHS 7 patients’ mean age was older: 74 years for males and 77 years for females. Younger individuals were the majority in MHS 1, 2 and 3, where the mean age rose for both genders in MHS 4, 5, 6 and 7, to lower once more in MHS 8 and 9.

The mean TTO score was 0.73, with a standard deviation of 0.32. The average EQ VAS was 64, with a standard deviation of 22.61. We found that the mean values of the TTO score and EQ VAS lowered when morbidity increased, which were 0.93 and 80.32 for MHS 1, and 0.49 and 53.13 for MHS 9, respectively (Table 1).

**Fig. 1:**
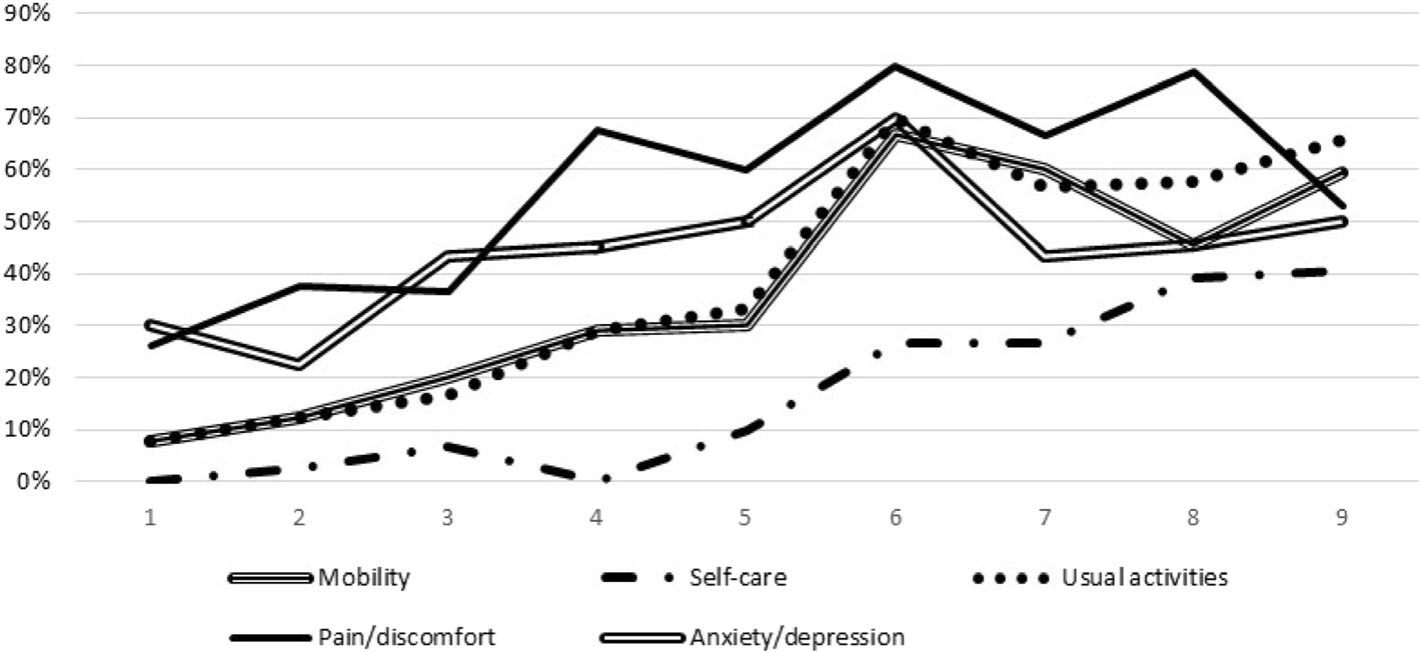
Percentages of health problems (moderate or severe problems) in the five EQ-5D dimensions in the nine MHS CRG

According to the HRQL questionnaire dimensions, 86 individuals in the sample had no health problem for any of the five dimensions, which represents 28%. The most referred to dimension was “pain/discomfort” with 54%, which had 42% of moderate problems and 11% of severe problems. The second most referred to dimension was “anxiety/depression” with 42%, of which 34% indicated a moderate health problem and 8% a severe one. In general terms, the least referred to health dimension was “self-care” with 15%, and only 2% indicated a severe health problem. When we differentiated the MHS (Fig. 1), we found more individuals were affected in the HRQL (moderate or severe problems) when morbidity increased for all the dimensions. The MHS 6 patients were those with more health problems for all the dimensions, except for “self-care”, whose severest health problem was in MHS 8 and 9.

The linear regression models, with statistically significant results, explained how the CRG system, age and gender influenced the HRQL. They are represented in Table 2. In the three models that explained the TTO score, an initial value was taken, which came close to 1 (from 0.93 to 0.99 of the intercept), and corresponded to the better health state patient’s HRQL. Age (Model 1) explained 4% of the TTO score with a negative coefficient, in such a way that when age increased, HRQL perception lowered by 0.004 points per year of life. The MHS (Model 2) explained 17% of HRQL variability. All the MHS had negative coefficients that grew with morbidity; thus the higher the morbidity, the lower the TTO score value. Gender (Model 3) had an impact only when it was associated with morbidity, which jointly explained 21%, and had a negative coefficient. This scenario indicates that the female gender obtained worse EQ-5D-3 L results (a mean of 0.10 points less than males).

**Table 2:** Multiple linear regression models that explain the EQ-5D-3 L utility score and EQ VAS

Variables	EQ 5D 3 L TTO score beta coefficient (95%CI)				EQ VAS beta coefficient (95%CI)	
	Model 1	Model 2	Model 3		Model 4	
Intercept	0.95	0.93	0.99		80.32	
Age	−0.004***					
Gender			−0.10	**		
MHS CRG						
1. Healthy (control variable)						
2. History of significant acute disease		−0.06	−0.05		−10.57	*
3. Single minor chronic disease		−0.12	−0.14	*	−14.49	**
4. Minor chronic diseases in multiple organ systems		−0.16*	−0.15	*	−13.87	**
5. Significant chronic disease		−0.18**	−0.20	**	−18.32	***
6. Significant chronic diseases in multiple organ systems		−0.35***	−0.36	***	−24.49	***
7. Dominant chronic disease in 3 or more organ systems		−0.33***	−0.35	***	−23.32	***
8. Dominant/Metastatic malignancy		−0.33***	−0.36	***	−28.65	***
9. Catastrophic		−0.44***	−0.47	***	−27.20	***
Adj. R^2^	0.04	0.17	0.21		0.15	
F-test	14.76***	9.73***	9.72	***	7.88	***

*MHS CRG*: Main Health Status Clinical Risk Groups

*TTO score*: Time Trade-Off score

*EQ VAS*: EQ Visual Analogue Scale

*p < 0.05, **p < 0.01, ***p < 0.001 by chi-square for the difference in the proportion of respondents referring to any problem (moderate or severe problem) across the variables

For EQ VAS, only the CRG system (Model 4) explained 15% of those with health problems according to the EQ VAS. The model’s intercept was 80.32, which came close to 100, namely the best imaginable health state. In Model 4, all the MHS also had negative coefficients that increased with morbidity; thus the higher morbidity was, the lower the HRQL perception values became. It is noteworthy that in Model 2, the coefficients of MHS 2 and 3 were not significant, which indicates that there were no differences in the TTO score with MHS 1. The same happened in Model 3 with the coefficient of MHS 2. However, the coefficient significantly increased in all the models (2, 3 and 4) when moving from MHS 5 to MHS 6, and all the MHS 6 coefficients were always higher than those of MHS 7.

When we analysed the influence of each MHS individually in all five dimensions (Table 3), in MHS 1 and 2, all the Odds Ratio were always significant and less than 1. This means that the MHS 1 and 2 individuals were more likely to not have problems in any dimension than the other MHS. Specifically for MHS 1, we noted this likelihood was 7.69-fold (1/0.13) for “mobility”, 8.33-fold for “usual activity”, 4.17-fold for “pain/discomfort” and 1.92-fold for “anxiety/depression”. For MHS 3, 4 and 5, no relation appeared between MHS and HRQL because the Odds Ratios were not significant.

**Table 3:** Binary logistic regression between the five dimensions and the MHS

Main Health status clinical risk groups	Sample *n* = 306 n	Mobility	Self-Care	Usual activities	Pain/discomfort	Anxiety/depression
		Odds Ratio	Odds Ratio	Odds Ratio	Odds Ratio	Odds Ratio
		95%CI	95%CI	95%CI	95%CI	95%CI
1. Healthy	50	0.13***	0	0.12***	0.24***	0.52*
		[0.05, 0.38]		[0.04, 0.35]	[0.12, 047]	[0.27, 0.99]
2. History of significant acute disease	40	0.24**	0.12*	0.22**	0.46*	0.34**
		[0.09, 0.62]	[0.02, 0.89]	[0.08, 0.57]	[0.23, 0.92]	[0.16, 0.75]
3. Single minor chronic disease	30	0.45	0.36	0.32*	0.46	1.02
		[0.18, 1.13]	[0.08, 1.55]	[0.12, 0.86]	[0.21, 1.00]	[0.48, 2.19]
4. Minor chronic diseases in multiple organ systems	31	0.76	0	0.69	1.91	1.11
		[0.34, 1.72]		[0.31, 1.57]	[0.87, 4.21]	[0.53, 2.35]
5. Significant chronic disease	30	0.80	0.57	0.87	0.48	1.38
		[0.35, 1.82]	[0.17, 1.96]	[0.39, 1.92]	[0.61, 2.84]	[0.65, 2.93]
6. Significant chronic diseases in multiple organ systems	30	4.49***	2.15	4.82***	3.83**	3.52**
		[2.02, 10.01]	[0.89, 5.15]	[2.12, 10.95]	[1.52, 9.66]	[1.55, 7.97]
7. Dominant chronic disease in 3 or more organ systems	30	3.26**	2.15	2.53*	1.81	1.02
		[1.50, 7.06]	[0.89, 5.15]	[1.18, 5.44]	[0.82, 4.00]	[0.48, 2.19]
8. Dominant/Metastatic malignancy	33	1.69	4.42***	2.67**	3.58**	1.13
		[0.82, 3.52]	[2.02, 9.67]	[1.28, 5.57]	[1.50, 8.53]	[0.55, 2.33]
9. Catastrophic	32	3.20**	4.67***	3.90**	0.97	1.38
		[1.50, 6.76]	[2.12, 10.29]	[1.80, 8.45]	[0.46, 2.01]	[0.66, 2.88]

*MHS CRG* Main Health Status Clinical Risk Groups

*CI* confidence interval

*p < 0.05, **p < 0.01, ***p < 0.001 by chi-square for the difference in the proportion of respondents referring to any problem (moderate or severe problem) across the variables

The Odds Ratio values for MHS 6, 7, 8 and 9 were always above 1 in all the dimensions, which suggests more likelihood of having problems in the dimensions. Indeed the MHS 6 individuals were more likely than all the others to have “mobility” (4.49-fold), “usual activity” (4.82-fold), “pain/discomfort” (3.83-fold) and “anxiety/depression” (3.52-fold) problems. However in the “self-care” dimension, the greater likelihood of having health problems was found for the MHS 9 and 8 patients (4.67-fold and 4.42-fold, respectively).

When we jointly analysed the impact of multimorbidity, age and gender (Table 4) on the HRQL dimensions, differences started appearing in some dimensions (“usual/activities” and “pain/discomfort”) in MHS 4 and in other dimensions (“mobility” and “anxiety/depression”) in MHS 5.

**Table 4:** Logistic regression models of the effect of MHS, age and gender on the “moderate/severe problems” for each dimension of HRQL

Variables		Mobility	Self-Care	Usual	Pain /	Anxiety /
				Activities	Discomfort	Depression
	Sample	Odds Ratio	Odds Ratio	Odds Ratio	Odds Ratio	Odds Ratio
	n = 306	95%CI	95%CI	95%CI	95%CI	95%CI
Intercept		0.01	0.00	0.03	0.15	1.07
Age		1.04***	1.01	1.01	1.01	0.98**
		[1.02, 1.07]	[0.98, 1.03]	[0.99, 1.03]	[1.00, 1.03]	[0.91, 0.99]
Gender		1.65	2.03	2.00*	1.40	1.28
		[0.93, 2.93]	[0.99, 4.16]	[1.12, 3.57]	[0.83, 2.35]	[0.77, 2.12]
MHS CRG						
1. Healthy	50					
2. History of significant acute disease	40	1.74	–	1.54	1.69	0.61
		[0.42, 7.23]	–	[0.38, 6.19]	[0.68, 4.19]	[0.23, 1.62]
3. Single minor chronic disease	30	3.49	–	2.72	1.80	1.92
		[0.86, 14.13]	–	[0.66, 11.21]	[0.67, 4.85]	[0.73, 5.06]
4. Minor chronic diseases in multiple organ systems	31	3.09	0.91	4.06*	5.14**	2.60
		[0.82, 11.66]	[0.00, 0.00]	[1.10, 15.06]	[1.89, 14.01]	[0.98, 6.92]
5. Significant chronic disease	30	4.19*	–	6.18**	4.24**	3.04*
		[1.10, 15.94]	–	[1.68, 22.70]	[1.58, 11.37]	[1.14, 8.08]
6. Significant chronic diseases in multiple organ systems	30	15.97***	–	25.07***	9.75***	8.23***
		[4.30, 59.29]	–	[6.67, 94.28]	[3.18, 29.89]	[2.88, 23.53]
7. Dominant chronic disease in 3 or more organ systems	30	7.90**	–	13.39***	4.37**	3.50*
		[2.09, 29.85]	-]	[3.47, 51.76]	[1.49, 12.76]	[1.20, 10.20]
8. Dominant/Metastatic malignancy	33	6.78**	–	16.95***	9.65***	3.15*
		[1.89, 24.38]	–	[4.64, 61.85]	[3.25, 28.66]	[1.16, 8.51]
9. Catastrophic	32	14.59***	–	24.69***	3.05*	3.41*
		[4.03, 52.84]	–	[6.72, 90.73]	[1.16, 8.1]	[1.28, 9.04]
Chi squared		85.02	69.18	83.35	50.64	30.09
Cox & Snell’s R^2^		0.24	0.20	0.24	0.15	0.09
Nagelkerke’s R^2^		0.34	0.35	0.33	0.20	0.13
Global percentage correctly classified						
		75.8	84.3	75.5	67.3	68.3

*MHS CRG* Clinical Risk Groups

*CI* confidence interval

R^2^: coefficient of determination

*p < 0.05, **p < 0.01, ***p < 0.001 by chi-square for the difference in the proportion of respondents referring to any problem (moderate or severe problem) across the variables

The “usual activities” dimension presented the largest differences among the MHS, with a 25-fold greater likelihood for the patients in MHS 6 and 9 compared to the “healthy” population, a 16.95-fold more likelihood in the MHS 8 individuals and a 13.39-fold greater one in the MHS 7 subjects.

No MHS influenced the “self-care” dimension. The highest coefficients were obtained in MHS 6 for all the dimensions, which entails worse HRQL.

## Discussion

One of our main findings was to determine the EQ-5D utility values for each morbidity status according to the CRG classification. The study also showed an association between the HRQL dimensions and the established multimorbidity statuses. The discussion of the results thus focused on comparing our findings with those reported in similar studies according to three main points: the method to stratify the population into multimorbidity levels, the usefulness of the CRG classification system, age and gender to determine HRQL scores, and the association between multimorbidity and the degrees of health problems in the HRQL dimensions. All this was done by addressing the health care offered from primary-care to patients with chronic diseases.

The distribution of the general population into health districts by MHS was similar to that obtained in previous studies conducted in the Valencian Community [14, 17, 42], but differed from those done in other Spanish Autonomous Communities. In our study, the population classified in MHS 1 (“healthy” or “non-users”) represented 53.9%, which differed from 61% in the Spanish Autonomous Communities of Madrid [10] or from 67% in the Spanish Autonomous Communities of Catalonia [8]. This was due to the gap that appears when implementing information systems [43, 44]. Following up the prevalence of chronic diseases is a good indicator to know and monitor their degree of implementing diagnosis codes [42].

The mean age (39.2 years) and gender distribution (49% males, 51% females) of our study population were similar to those reported in other studies [45]. Mean age increased as MHS advanced [8, 45] and mean age exceeded 50 years from MHS 4 (54 years), a previous MHS to the usual one. This corroborates that multimorbidity starts at increasingly earlier ages, and this finding agrees with the consulted bibliography [10–14]. This allowed us to detect the population with multimorbidity in the patients classified as MHS 4 and onwards.

One main characteristic of our work was to study the HRQL in a representative sample of the general population, unlike other studies that have focused on patient groups with specific diseases [13, 27, 32, 40, 41, 46] or on older populations [9, 40]. A generic measuring instrument was used, EQ-5D-3 L, as it can suitably serve to analyse non-specific diseases and to compare groups of individuals with different medical conditions. In Alberta (Canada) [22] used the EQ-5D questionnaire to observe an association that linked chronic diseases and HRQL, hospitalisations and emergencies. A recent study [47] in Portugal has shown a relationship between HRQL and multimorbidity in primary-care. As in our study, the number of chronic conditions was related with worse QoL. However, the results were not comparable because the above-cited authors measured QoL with their own measuring instruments and they focused on determining any unfilled requirements that affected QoL. Another study [31] found that HRQL lowered as morbidity measured by the CIRS (Cumulative Illness Rating Scale) increased.

No differences were found among MHS 1, 2 and 3 in their influence on HRQL for all the dimensions, the TTO score and EQ VAS. Differences started to appear in MHS 4, where multimorbidity began, which demonstrates the association between morbidity and HRQL. The most affected HRQL dimensions (moderate/severe problems) of our sample were “pain/discomfort”, followed by “anxiety/depression” (54 and 42%, respectively), which also occurred in other studies [22, 30]. We noted that the MHS 6 patients, with mean ages of 56–65 years, presented more health problems for all the dimensions, except for “self-care”, which appeared in MHS 8 and 9 patients, unlike the MHS 7 patients with older mean ages (74 and 77 years). The likelihood of having more health problems for all the dimensions was greater in MHS 6, except for the “self-care” dimension.

We obtained the same results with the linear regression analysis when connecting HRQL (TTO score and EQ VAS) with belonging to a given MHS, age and gender. MHS had the strongest influence on HRQL (17 and 15% on the TTO score and EQ VAS, respectively, vs. 4% on age). The regression coefficients of Model 3 can be used as a utility value to perform a cost-effectiveness analysis, as previous studies have done for other scales [33].

These adjusted R^2^ values were similar to those obtained in another study [9], with 17.1 and 17.7%, respectively. The remaining HRQL variability could be given by other factors that this study did not contemplate; for instance: patients supported by their family, economic income, occupational status, job type, place of residence, etc., which could result in many personal situations and could, therefore, lead to a different HRQL perception. We found that the lower the morbidity in younger patients, the worse HRQL became, just as other studies have reported [9, 22], because the low HRQL expectations in an older population and the ability to adapt to life styles mean that HRQL perception is better despite morbidity being higher [13].

We also observed that, regardless of MHS, being female implied a worse HRQL, where the affected dimension was “usual activities”. This provides previous studies about a worse perceived HRQL by the female gender with further information [30, 48].

As our study work was conducted with the populations of all the MHS, the next step will be to replicate these analyses with the main diseases that affect the general population, like diabetes [13] or mental diseases, which can more strongly influence HRQL than clinical conditions can. It would also be interesting to include socio-economic aspects in the future to supplement information on co-morbidity and demographic aspects, which have been studied before in other works [41].

Some limitations need to be considered. Although a modest sample size was used, the errors reported herein did not significantly differ from those reported in other studies [33].

Although few individuals were included in the population integrated into the groups with higher morbidity (8 and 9), a sample was obtained with a homogeneous number of individuals in all the groups, with 306 patients in all. Other similar studies [31, 32, 34] used smaller samples, with 238 patients and 83 patients, respectively. Selecting sample with subjects aged over 18 provided us with a mean age of 53 years, which is older than that of the general population (39.2 years). However with each MHS (see Table 1), individuals’ mean age in the sample came close to the population’s values, except in the healthy group.

The results herein generated from a small sample would be useful to validate studies with a large sample.

## Conclusions

This work demonstrates that the multimorbidity groups obtained by the CRG classification system can be used as an overall indicator of HRQL. These scores can be used for health policy decisions based on cost-effectiveness to estimate incremental QALY using routine e-health data.

One very useful tool for health planning and healthcare activities is having a system that adjusts risks based on clinical variables to help identify those patients with several diseases, have a higher disease burden and are at higher clinical risk. Their inclusion in patients’ electronic health records as warnings is currently a practice in some areas of Spain. By adding other aspects, like the impact on HRQL and socio-economic variables to this purely clinical information, will enable to qualitatively take a step forward in health care, and will doubtlessly prove a tool to help health professionals to improve the results obtained in overall patient care. This is particularly relevant for electronic health records which are more strategically willing to improve socio-health care integration and coordination.

## Abbreviations

CRG: Clinical Risk Groups
EQ VAS: EQ Visual Analogue Scale
HRQL: Health-Related Quality of Life
MHS: Mean health statuses
QALY: quality-adjusted life year
R^2^: Coefficient of determination
TTO: Time Trade-Off
